# Munc18-Bound Syntaxin Readily Forms SNARE Complexes with Synaptobrevin in Native Plasma Membranes 

**DOI:** 10.1371/journal.pbio.0040330

**Published:** 2006-09-26

**Authors:** Felipe E Zilly, Jakob B Sørensen, Reinhard Jahn, Thorsten Lang

**Affiliations:** 1 Department of Neurobiology, Max Planck Institute for Biophysical Chemistry, Göttingen, Germany; 2 Department of Membrane Biophysics, Max Planck Institute for Biophysical Chemistry, Göttingen, Germany; Princeton University, United States of America

## Abstract

Munc18–1, a protein essential for regulated exocytosis in neurons and neuroendocrine cells, belongs to the family of Sec1/Munc18-like (SM) proteins. In vitro, Munc18–1 forms a tight complex with the SNARE syntaxin 1, in which syntaxin is stabilized in a closed conformation. Since closed syntaxin is unable to interact with its partner SNAREs SNAP-25 and synaptobrevin as required for membrane fusion, it has hitherto not been possible to reconcile binding of Munc18–1 to syntaxin 1 with its biological function. We now show that in intact and exocytosis-competent lawns of plasma membrane, Munc18–1 forms a complex with syntaxin that allows formation of SNARE complexes. Munc18–1 associated with membrane-bound syntaxin 1 can be effectively displaced by adding recombinant synaptobrevin but not syntaxin 1 or SNAP-25. Displacement requires the presence of endogenous SNAP-25 since no displacement is observed when chromaffin cell membranes from SNAP-25–deficient mice are used. We conclude that Munc18–1 allows for the formation of a complex between syntaxin and SNAP-25 that serves as an acceptor for vesicle-bound synaptobrevin and that thus represents an intermediate in the pathway towards exocytosis.

## Abstract 2

The authors take a fresh look at the role of Munc-18, an SM protein, in vesicle fusion and find that it facilitates the formation of fusion-competent SNARE complexes by binding to syntaxin.

## Introduction

Sec1/Munc18 (SM) proteins comprise a small family of cytoplasmic proteins that play a pivotal role in intracellular membrane fusion. They are structurally highly conserved in evolution, and each SM protein is specialized for a single or a small group of trafficking steps. SM proteins of evolutionarily distant species that are involved in the same trafficking steps are capable of replacing each other whereas within one organism, different SM proteins show no functional redundancy (reviewed by [1]).

Membrane fusion in the secretory pathway is catalyzed by SNAREs (soluble N-ethylmaleimide–sensitive factor attachment protein receptors), which are represented by a superfamily of small, membrane-anchored proteins. For effecting fusion, SNAREs located in opposing membranes assemble into tight complexes and force the membranes into close apposition, initiating the merger of bilayers. Assembly is mediated by a stretch of 60–70 amino acids, termed SNARE motif, which is characteristic for all SNAREs and usually located adjacent to the C-terminal transmembrane domains. SNARE motifs are grouped into four subfamilies that are referred to as Qa-, Qb-, Qc-, and R-SNARE motifs, respectively. SNARE complexes consist of structurally conserved bundles of four α-helices, in which each helix is contributed by a SNARE motif belonging to a different subfamily (for review see [2–4]).

Most available data suggest that SM proteins exert their function by acting upon SNAREs. Best documented is the direct interaction between SM proteins and a selected set of syntaxins (Qa-SNAREs). Furthermore, deletion of some SM proteins is associated with a concomitant reduction in Qa-SNARE levels and vice versa, and strong genetic interactions have been observed between the two protein classes [5–10]. Despite major efforts, however, it has been impossible to explain coherently how SM proteins function at the molecular level. Most importantly, the binding mode between SM and SNARE proteins is not conserved [1,11]. All Qa-SNAREs contain separately folded N-terminal domains, represented by bundles of three antiparallel α-helices that are connected to the SNARE motif by short linkers [12]. Whereas the SM proteins Sly1p and Vps45p bind only to the N-terminal tips of their respective Qa-SNAREs Sed5p and Tlg2p [13–15], respectively, binding of syntaxin 1 to the SM protein Munc18–1 involves both N- and C-terminal regions and requires the N-terminal domain to be folded back on the SNARE motif (closed conformation) [16]. This difference in binding modes has a profound impact on the ability of the corresponding Qa-SNARE protein to interact with its respective partner SNAREs. Sly1p or Sec1p do not impede the ability of Sed5p or Sso1/2p, respectively, to enter SNARE complexes [15,17]. By contrast, Munc18–1 binding to syntaxin 1 completely prevents the formation of SNARE complexes, and Munc18–1 needs to dissociate before syntaxin 1 can bind to its SNARE partners synaptobrevin 2 (also referred to as VAMP 2 [vesicle-associated membrane protein 2]) and SNAP-25 (synaptosome-associated protein of 25 kDa) [18,19]. The remarkable divergence in Qa-SNARE–SM protein interactions was underscored by the crystal structures of the corresponding complexes: In the complex of Sly1p and Sed5p, only the N-terminal peptide of Sed5p participates in the protein–protein interaction by making a local contact with a small groove on the outer surface of Sly1p [20]. In stark contrast, in the corresponding Munc18–1/syntaxin 1 complex, the latter resides closed in a conserved cleft of Munc18–1, with numerous crystal contacts between Munc18–1 and both the N-terminal domain and the SNARE motif of syntaxin [16].

Unlike its ubiquitously expressed relatives Munc18–2 and Munc18–3 [21,22], Munc18–1 and its SNARE-partners function in exocytosis of neurons and neuroendocrine cells. Considering that interaction between SNAREs is essential for exocytosis, and that Munc18–1 binding to syntaxin precludes any interaction with its partner SNAREs in vitro, one would expect that Munc18–1 serves as a negative regulator of exocytosis. However, this view cannot be reconciled with the phenotype of Munc18–1–deficient animals or with experiments involving Munc18–1 overexpression. For instance, in mice and *Caenorhabditis elegans,* Munc18–1 deletion leads to a complete block of neurotransmitter release [5,9]. Although the morphology of synapses is largely normal, syntaxin levels are reduced [5]. Conversely, even massive overexpression of Munc18–1 has no negative effect on exocytosis in most systems [6,23–27] (for exception see [28]). Together, these and other studies strongly suggest that Munc18–1 has an activating rather than an inhibitory role in exocytosis.

Munc18–1 not only binds to syntaxin, but also interacts with other proteins such as Mint [27,29]. Thus it is conceivable that syntaxin binding merely serves as a recruiting mechanism aimed at increasing the local concentration, and that the rate-limiting effect of the protein on exocytosis is exerted on the SNAREs by an indirect mechanism. For the Qa-SNARE Sed5p, it has recently been shown that removal of the N-terminal peptide responsible for Sly1p binding has no functional consequences [30], supporting the view that Qa-SNARE binding is not part of an SM protein's essential function. On the other hand, binding of Munc18–1 to syntaxin 1 is needed for exocytosis. In most cases, overexpression of Munc18–1 mutants whose binding to syntaxin is impaired negatively affects exocytosis [24,25,27]. Thus it is not possible at present to reconcile the fact that Munc18–1 only binds to the closed and inactive form of syntaxin 1 with the large body of evidence documenting that Munc18–1 function is essential for exocytosis to proceed.

In the present study, we have taken a fresh look at the role of Munc18–1 in the formation of SNARE complexes. We used inside-out plasma membranes from phaeochromocytoma (PC12) cells, which allow direct biochemical access to the release apparatus while retaining competence for regulated exocytosis [31,32]. Our results show that Munc18–1 is bound to the membrane and concentrated at sites of docked vesicles. As expected, the main binding site was identified as syntaxin 1, and we confirmed that the ability of syntaxin to form a closed conformation is necessary for efficient recruitment of Munc18 to the plasma membrane. Surprisingly, however, we found that membrane-bound Munc18–1 can be directly displaced by synaptobrevin in a reaction that requires the presence of SNAP-25, i.e., that Munc18–1 binding to syntaxin does not prevent SNARE complex formation in an intact membrane. We conclude that Munc18–1 stabilizes a labile and half-closed state of syntaxin 1, probably associated with SNAP-25, that may represent a physiological intermediate in the formation of fusion-competent SNARE complexes.

## Results

To characterize association of Munc18–1 with the plasma membrane of secretory cells, we prepared inside-out sheets of membranes using ultrasound treatment of intact PC12 cells. We have shown previously that granules remain attached to these sheets and are able to undergo calcium-regulated exocytosis ([31,32]). Furthermore, syntaxin was shown by this and other techniques to be concentrated in cholesterol-dependent microdomains that determine the sites at which secretory granules dock and fuse [33,34].

When membrane sheets were immediately fixed and stained for Munc18–1, fluorescence labeling was concentrated in numerous spots (Figure 1A, right panel). When the sheets were incubated in buffer at 37 °C for increasing periods of time before fixation, Munc18–1 slowly dissociated from the membrane as indicated by a gradual loss in immunoreactivity (Figure 1B and 1C), whereas syntaxin immunofluorescence, for example, was unchanged (unpublished data). Note that under our experimental conditions, no re-binding occurs because dissociated Munc18–1 is diluted in a large volume of buffer. To characterize the nature of the binding site, the sheets were incubated for 10 min with 10 μM of the light chains of clostridial neurotoxins that cleave syntaxin (botulinum neurotoxin (BoNT) C1), SNAP-25 (BoNT/E), or synaptobrevin (tetanus toxin), followed by immediate fixation and immunostaining for Munc18–1. All values were normalized to the 10-min control value. As shown in Figure 1D, Munc18–1 was specifically removed by BoNT/C1 whereas the other toxins were ineffective. The degree of cleavage was lower than the degree of syntaxin cleavage (Figure 1E). It is possible that a small pool of Munc18–1 is bound independently of syntaxin although it cannot be excluded that the toxin has a preference for uncomplexed syntaxin (the cleavage site is close to the transmembrane domain between residues 254 and 255 [35]). As control, the sheets were incubated with a BoNT/C1 mutant that was rendered inactive by a single amino acid substitution in the catalytic center [36]. No effect on Munc18–1 binding was observed. Together, these data confirm that the majority of Munc18–1 is membrane bound via the SNARE syntaxin 1. Furthermore, we tested whether Munc18–1 is associated with docked granules in our preparation. For this purpose, we prepared sheets from PC12 cells that were transfected with NPY-GFP as a marker for secretory granules [37]. A very high (at least 70%) degree of co-localization was observed between labeled granules and Munc18–1 clusters (Figure 2), as already suggested by a previous report [38].

**Figure 1:**
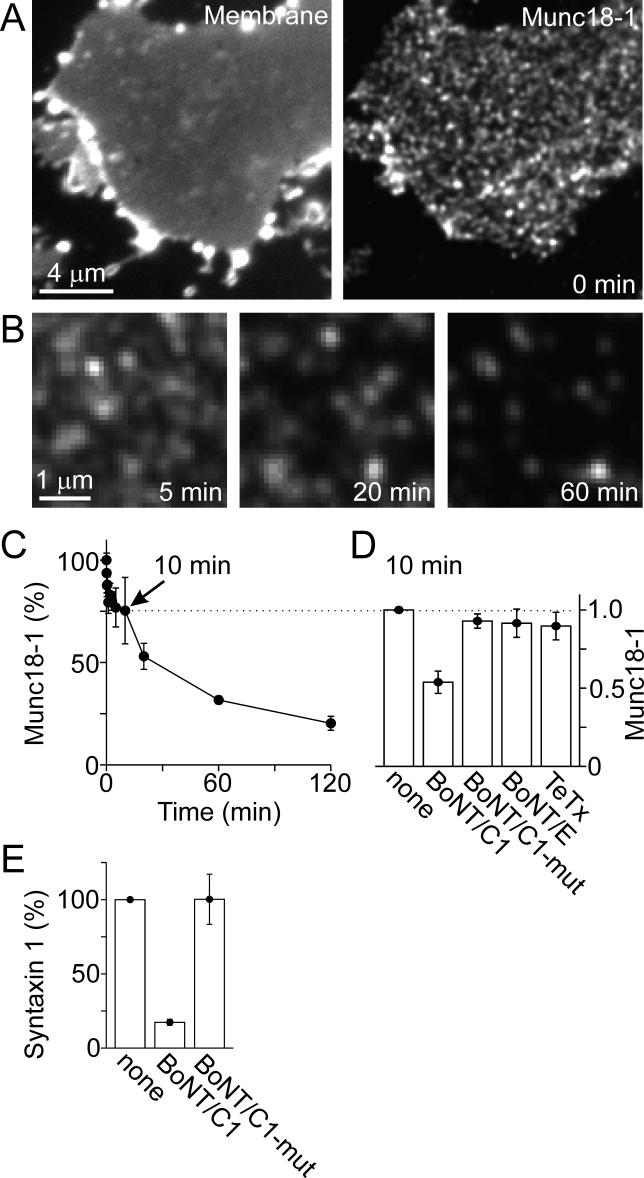
Characterization of Munc18–1 Complexes in Native Plasma Membranes (A) Munc18–1 is concentrated in microdomains in inside-out plasma membrane sheets from PC12 cells. Membrane sheets were fixed with paraformaldehyde immediately after preparation, followed by immunostaining for Munc18–1 (right panel). Integrity of the membrane was confirmed using counterstaining by the lipophilic dye TMA-DPH (left panel). (B) and (C) Time-dependent dissociation of Munc18–1 from the membrane sheets. The experiments were carried out as in (A), but between preparation and fixation, the sheets were washed with buffer for varying time periods (as indicated), resulting in a gradual decrease of Munc18–1 immunostaining intensity over time (C). Values were related to the immediately fixed condition which was set to 100% (*n* = 3–6 independent experiments for each data point, values are given as mean ± standard error of the mean (SEM); for details see Materials and Methods). (D) Sensitivity of the Munc18–1 acceptor to SNARE-cleaving light chains of clostridial neurotoxins. Membrane sheets were incubated for 10 min (see also arrow in C) in the absence (value used for normalization) or presence of 10 μM light chains as indicated, followed by fixation and immunostaining for Munc18–1. BoNT/C1 cleaves syntaxin 1A (BotNT/C1-mut, a cleavage-inactive BoNT/C1-point mutant was used as a control), BoNT/E cleaves SNAP-25, and synaptobrevin 2 is sensitive to TeTx. Values are given as mean ± SEM. *n* = 8, paired *t*-test analysis none:BoNT/C1 *p* < 0.0005. (E) Cleavage efficiency of BoNT/C1. Membrane sheets were incubated for 10 min in the absence (set to 100%) or presence of 10 μM light chains of BoNT/C1 or BoNT/C1-mut, followed by fixation and immunostaining for syntaxin 1. Values are given as mean ± SEM. n = 3.

**Figure 2:**
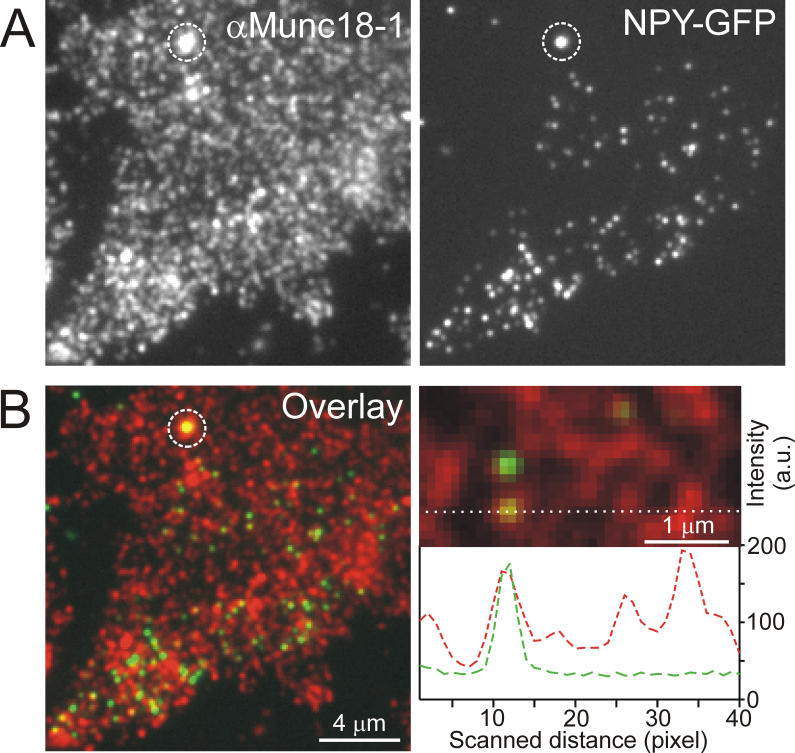
Docking of Secretory Granules to Plasmalemmal Domains Enriched in Munc18–1 (A) Plasma membrane sheet generated from a PC12 cell expressing the secretory granule marker NPY-GFP. Left, immunostaining for Munc18–1 (red channel); right, plasma membrane–docked, GFP-filled secretory granules (green channel). Circle indicates a fluorescent bead visible in all channels acting as a spatial reference for vertical shifts occurring during filter change. (B) Left, overlay from images shown in (A); right, magnified view from overlay. Linescans were placed through the centers of individual secretory granules (174 granules from ten membrane sheets were analyzed; for example, see dotted line), and granules were rated to be associated with a Munc18–1–rich domain when both signals had a maximum to within two pixels. Random co-localization was determined on mirrored images and subtracted (for details see Materials and Methods), resulting in 70% specific co-localization of granules with Munc18–1 domains.

Next, we tested whether syntaxin needs to be capable of adopting a closed conformation in order to recruit Munc18–1 to the plasma membrane. For this purpose, we took advantage of a previously characterized mutant form of syntaxin (open syntaxin, carrying the mutations L165A and E166A) which cannot form a closed conformation and which consequently shows a greatly reduced affinity for Munc18–1 [39]. GFP-tagged versions of both wild-type and open syntaxin were co-transfected with myc-tagged Munc18–1 in PC12 cells, followed by the preparation of membrane sheets, fixation, and immunolabeling for myc-tagged Munc18–1. As shown in Figure 3, more Munc18–1 is retained on the membrane when wild-type syntaxin and Munc18–1 levels are increased. However, this increase in Munc18–1 recruitment was markedly reduced when open syntaxin was expressed instead of wild-type syntaxin, documenting that Munc18–1 binding requires the ability of syntaxin to adopt a closed conformation.

**Figure 3:**
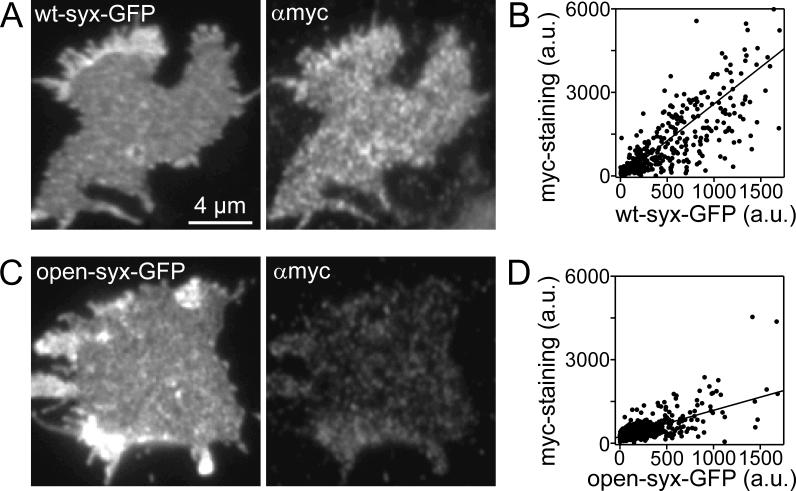
Munc18–1 Membrane Recruitment Requires the Closed Conformation of Syntaxin Co-overexpression of myc-tagged Munc18–1 together with GFP-tagged syntaxin 1 (A) and (B) or an open mutant of syntaxin 1 (C) and (D). Membrane-recruited overexpressd Munc18–1 was selectively visualized on the background of endogenous Munc18–1 by immunostaining for the myc-tag. For each condition, the level of overexpressed syntaxin and of overexpressed, recruited Munc18–1 were determined for individual membrane sheets, plotted against each other, and linearly fitted (B) and (D). The ratio of the slopes wild-type (wt) syntaxin:open syntaxin was determined to be 2.1. The graphs contain data points from 411 membrane sheets for wt syntaxin 1/Munc18–1 and 417 for open syntaxin 1/Munc18–1 obtained from ten independent experiments.

The data described so far show (1) that Munc18–1 is bound to the membrane via syntaxin 1, and (2) that for Munc18–1 binding, syntaxin must be able to form a closed conformation. These findings are in line with solution experiments, and they are also in agreement with previous findings using transfections of intact cells [18,39–41]. The question then arises whether Munc18–1 indeed inactivates syntaxin as it does in solution, or whether in intact and exocytosis-competent membrane sheets, it binds to a different conformational state that may be an intermediate in the pathway toward formation of SNARE complexes.

How can one test for this possibility? We have previously shown that in intact membranes, syntaxin can be readily driven into ternary SNARE complexes upon addition of recombinant synaptobrevin [42]. If Munc18–1 only binds to a completely closed syntaxin, this syntaxin pool would not be available for SNARE complex formation and no interaction with synaptobrevin is expected. If, however, Munc18–1–bound syntaxin 1 is half-closed and thus can interact with its partner SNAREs, addition of synaptobrevin is expected to recruit Munc18–1–bound syntaxin 1 into ternary SNARE complexes, leading to a synaptobrevin-induced dissociation of Munc18–1. We therefore tested if synaptobrevin is capable of driving off Munc18–1 from the membrane.

Membrane sheets were freshly prepared, and 10 μM recombinant synaptobrevin was added for 10 min, followed by fixation and immunostaining for Munc18–1. As shown in Figure 4A, addition of synaptobrevin led to a dramatic reduction in bound Munc18–1. Reduction was specific for the R-SNARE synaptobrevin since no effect was observed when syntaxin 1 or SNAP-25 was added. To further characterize synaptobrevin-induced displacement of Munc18–1, a time course experiment was performed. Displacement is rapid (Figure 4B), being almost completed after 10 min of incubation, and a small background remains, indicating that a fraction of the syntaxin molecules might adopt a closed conformation or is not accessible for synaptobrevin within the densely packed clusters.

**Figure 4:**
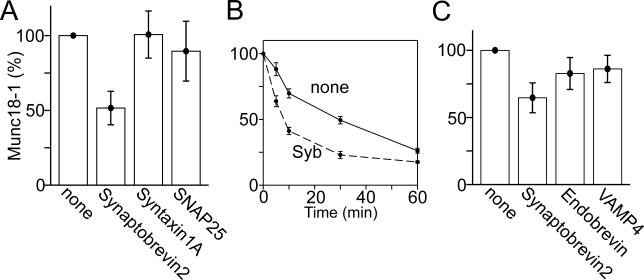
Synaptobrevin 2 Releases Munc18–1 from Complexed Syntaxin (A) Membrane sheets were incubated for 10 min in the absence (set to 100%) or presence of 10 μM of recombinant SNARE proteins as indicated, and Munc18–1 was quantified by immunofluorescence. Each experiment was performed six to seven times, values are given as mean ± standard deviation of the mean (SDM). Paired *t*-test analysis none:synaptobrevin 2 *p* < 0.0005 (*n* = 7); *t*-test synaptobrevin:SNAP-25 *p* < 0.005 (*n* = 6). (B) Incubation of membrane sheets with or without 10 μM synaptobrevin 2 for varying times, as indicated, followed by immunostaining for Munc18–1. Immunostaining intensity of immediately fixed membrane sheets was set to 100%. For each data point, four to six independent experiments were performed. Values are given as mean ± SEM. (C) Experiment as in (A), R-SNAREs were added during incubation as indicated. Values are given as mean ± standard deviation of the mean (SDM). Paired *t*-test synaptobrevin 2:none *p* < 0.00005 (*n* = 10), synaptobrevin 2:endobrevin *p* < 0.0005 (*n* = 10), and non-paired *t*-test synaptobrevin:VAMP4 *p* < 0.005 (*n* = 10 and 6).

We then tested whether Munc18–1 displacement is specific for synaptobrevin or whether other R-SNAREs are capable of substituting in the displacement reaction. Previous studies have shown that R-SNAREs such as endobrevin/VAMP8 can substitute for synaptobrevin both in complex formation as well as in liposome fusion experiments, with no significant difference in complex stability or assembly kinetics [43–46]. However, it has recently been suggested that the specificity of SNARE assembly may be increased in the presence of SM proteins [15], giving rise to the idea that SM proteins may be involved in the proofreading of SNAREs during complex formation; and as shown in Figure 4C, the R-SNAREs endobrevin and VAMP4 were less efficient in displacing Munc18–1.

We have shown previously that exogenous synaptobrevin recruits both endogenous syntaxin 1 and SNAP-25 into ternary SNARE complexes [42]. Consequently, synaptobrevin binding is largely prevented if either syntaxin or SNAP-25 is cleaved by the corresponding neurotoxins BoNT/C1 and BoNT/E [42]. However, BoNT/E does not release Munc18–1 from its membrane acceptor (Figure 1C), nor does it prevent synaptobrevin-induced Munc18–1 displacement under conditions in which approximately 90% of SNAP-25 is cleaved (unpublished data). There are several explanations for this finding: (1) the Munc18/syntaxin complex is associated with SNAP-25 in a manner that does not require the C-terminal SNARE motif or that renders SNAP-25 resistant to toxin cleavage, or (2) synaptobrevin-induced displacement of Munc18 does not involve any interaction with SNAP-25. To differentiate between these possibilities, we analyzed Munc18–1 displacement in membrane sheets obtained from primary cultured chromaffin cells derived from embryonic SNAP-25 knockout mice. Chromaffin cells from this developmental stage are fully functional, and in cells derived from SNAP-25 knockout mice, Ca-triggered exocytosis is abolished [47]. The amount of membrane-bound Munc18–1 was comparable between wild-type and knockout cells (Figure 5, and unpublished data), suggesting that SNAP-25 is not needed for Munc18–1 binding. Synaptobrevin was capable of displacing Munc18–1 as efficiently as in PC12 cells when sheets obtained from wild-type mice were used (Figure 5A and 5C). In contrast, no displacement was observed on membrane sheets from SNAP-25–deficient mice (Figure 5B and 5C). We conclude that synaptobrevin causes displacement of Munc18–1 from syntaxin in a reaction that requires the presence of SNAP-25, suggesting an intermediate acceptor complex for synaptobrevin that contains Munc18–1, syntaxin 1 and (most likely associated) SNAP-25.

**Figure 5:**
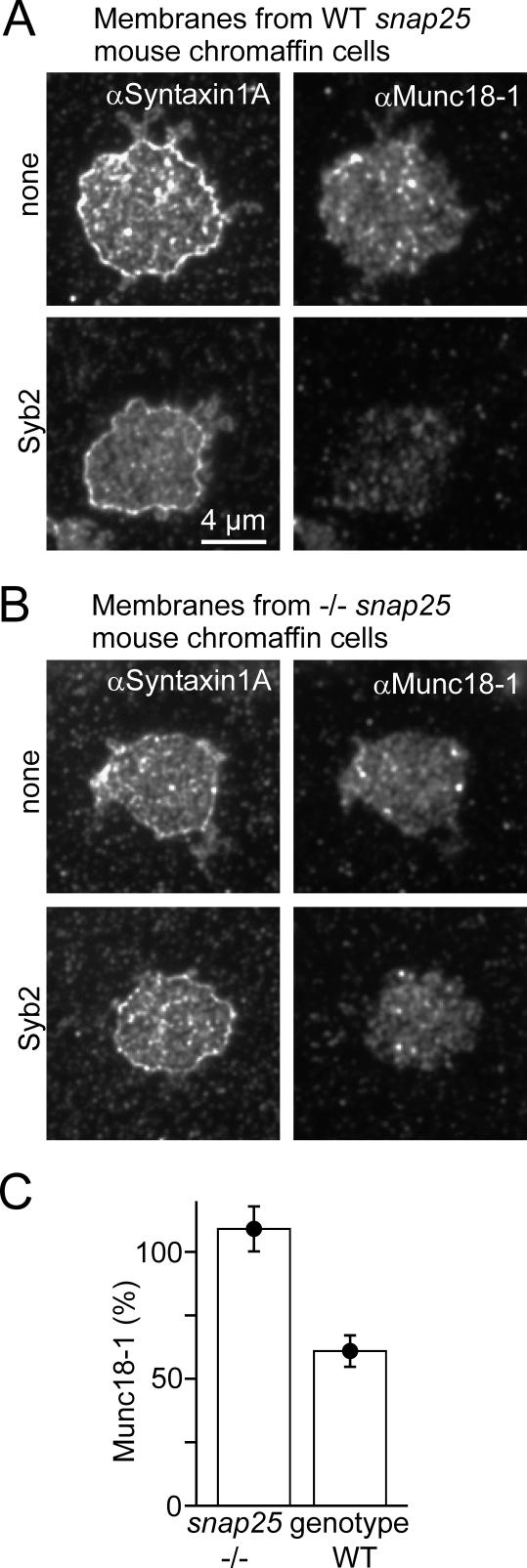
SNAP-25 Is Essential for Synaptobrevin 2–Triggered Munc18–1 Release Membrane sheets were prepared from mouse embryonal chromaffin cells (E16–E18), isolated from wild-type or *snap25* knockout animals. Membrane sheets were incubated for 10 min in the absence or presence of 10 μM synaptobrevin 2, fixed, and immunostained for Munc18–1. Syntaxin was also visualized by immunostaining in order to make sure that membrane sheets were generated from chromaffin cells and not from fibroblast cells, which are also present in the culture (for details see Materials and Methods). (A) and (B) Membrane sheets generated from cells isolated from wild-type (WT) (A) or knockout (B) animals. Left panels, syntaxin staining; right panels, Munc18–1 staining. Upper and lower panels in (A) and (B), absence or presence of synaptobrevin during incubation, respectively. (C) Quantification of Munc18–1 immunostaining intensities. For clarity, values are expressed as percentage relating to values obtained in the absence of synaptobrevin. For each condition, seven independent experiments were performed. Values are given as mean ± SEM. Statistical test. *n* = 7, paired *t*-test knockout:wt *p* < 0.005.

## Discussion

In the present study we have shown that in functionally intact plasma membranes, Munc18–1 binds to an intermediate conformation of syntaxin. Munc18–1–bound syntaxin 1 is capable of entering SNARE complexes with SNAP-25 and synaptobrevin, resulting in the dissociation of Munc18–1. Our findings thus provide direct biochemical support for a model in which Munc18 is involved in the formation of fusion-competent trans-SNARE complexes. This notion is in agreement with the assumed function of other SM proteins, strengthening the view that despite the remarkable differences in SM protein/Qa-SNARE binding, SM proteins may share a common molecular mechanism.

Although intensely studied, Munc18–1 has remained an oddity among SM proteins. Numerous studies have established that Munc18–1 binds exclusively to the closed conformation of syntaxin, and that closed syntaxin is completely inhibited in its ability to enter SNARE complexes (see, e.g., [16,18,19,39–41]). In contrast, the binding mode of other SM proteins such as Sly1p, Vps45p, and Sec1p does not prevent the respective Qa-SNAREs from forming complexes with their partner SNAREs. For instance, when Sly1p is bound to the Qa-SNARE Sed5p, kinetics of SNARE assembly is unchanged, and specificity for the cognate SNAREs is increased [15]. Furthermore, Sec1p has actually been shown to promote SNARE-mediated fusion of liposomes, providing direct biochemical evidence in support of an enhancing role of SM proteins in the formation of fusion-competent SNARE complexes [48].

Our data now show that Munc18–1 binds, and perhaps stabilizes, a conformation of syntaxin that is capable of forming SNARE complexes. Although neither the structure nor the precise composition of this intermediate complex is known, the following conclusions can be made: (1) syntaxin is neither completely closed, since it can bind to SNAREs, nor completely open, because Munc18–1 does not bind to a constitutively open mutant of syntaxin. In fact, the observed displacement of Munc18 can best be explained if synaptobrevin causes a partially open syntaxin to fully open as is required for SNARE-complex formation, causing Munc18–1 to dissociate (Figure 6). (2) SNAP-25 is associated with or at least readily available for the Munc18/syntaxin complex, but the nature of this mechanism is unclear. SNAP-25 is either resistant to BoNT/E cleavage in this complex, or else cleavage does not affect its function in the Munc18/syntaxin complex. In the latter case, only the N-terminal Qb-motif and the truncated C-terminal Qc-motif would participate in the complex. (3) The high degree of co-localization between granules and Munc18–1 suggests that the Munc18/syntaxin complex is needed at sites where granules dock and fuse, strengthening the view that the complex is an intermediate in the SNARE pathway towards fusion. This observation agrees with the localization of yeast SM protein Sec1p that is concentrated at the bud site (i.e., at the site where vesicles fuse) although the Qa-SNARE Sso1/2p is evenly distributed over the plasma membrane [17,49].

**Figure 6:**
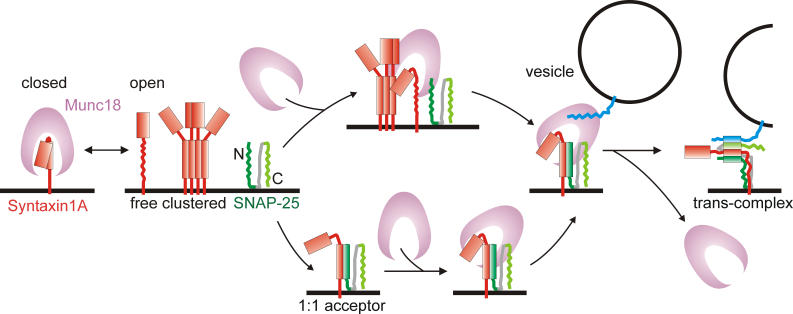
Model of Munc18–1 Function in the SNARE Assembly Pathway Munc18–1 binds to a partially closed conformation of syntaxin that is organized in clusters and that may (bottom branch) or may not (top branch) be associated with SNAP-25 at a 1:1 stoichiometry. After vesicle docking, synaptobrevin interacts with this complex, thereby displacing Munc18–1. Alternatively, SNAP-25 is not yet associated with the complex, and synaptobrevin binding is associated with the simultaneous recruitment of SNAP-25. As result, SNARE *trans*-complexes form, leading to exocytosis. It is possible that some syntaxin molecules that are freely diffusing in the membrane are capable of binding Munc18–1 with high affinity in a closed conformation (left) similar to that observed in solution, thereby preventing it from entering SNARE complexes. Such a pool could be reflected by Munc18–1 remaining membrane associated even after extensive washing periods.

Our data also shed new light on the status of syntaxin in the plasma membrane. We believe that syntaxin cannot assume a fully closed conformation, for the following reason. Although somewhat reduced in the presence of the transmembrane domain [50], closed syntaxin has a very high affinity for Munc18–1 (K_d_ = 6 nM [18]). Despite such high affinity, only a fraction of the cellular pool of Munc18–1 is membrane bound under steady-state conditions. Since PC12 cells contain approximately 20 times more syntaxin than Munc18–1 [27], such a large excess of binding sites should result in a quantitative recruitment of Munc18–1 to the membrane if binding were indeed of high affinity. Full closure of syntaxin may be prevented by its homo-oligomerization in clusters [51], or by a loose association with SNAP-25, resulting in a Munc18–1 binding site of reduced affinity. A lower affinity for Munc18–1 binding is also supported by our observation that Munc18–1 is washed out from the membrane within 15–30 min. Although the large majority can be readily washed out, it cannot be excluded that the minor fraction remaining bound after 120 min represents Munc18–1 bound with high affinity to fully closed syntaxin.

Is Munc18–1 binding necessary for syntaxin to form SNARE complexes? We have shown previously that syntaxin can be quantitatively driven into ternary SNARE complexes upon addition of recombinant synaptobrevin [42]. As outlined above, the Munc18–1–bound pool represents only a fraction of all syntaxin in the plasma membrane, and thus it is likely that syntaxin does not need Munc18–1 to form complexes. However, synaptobrevin added from the outside is conformationally not restricted like synaptobrevin on a secretory vesicle destined to dock and fuse. Thus it is conceivable that the syntaxin 1/SNAP-25 complex that is stabilized by Munc18–1 exhibits special features that favor interaction with vesicle-bound synaptobrevin (Figure 6), and that such an interaction might be even promoted by accessory factors. This would prevent uncoupling of SNARE complex formation and membrane fusion, as ternary SNARE complexes can form in principle also between only plasma membrane–associated SNAREs. This is consistent with the observation that in Munc18–1 knockout animals, the level of ternary SNARE complexes is largely increased [52].

In summary, the data presented here allow for reconciling some of the discrepancies between different SM proteins, which have prevented the development of a common molecular model. Furthermore, the data highlight again that neuronal SNAREs form highly labile intermediates on the pathway to fusion that show off-pathway reactions into dead-end complexes, such as the 2:1 complex between syntaxin 1 and SNAP-25 [53]. It is conceivable that the closed conformation of syntaxin represents a similar “off-pathway” conformation, which has hitherto occluded the molecular function of Munc18–1 in regulated exocytosis.

## Materials and Methods

### Cell culture.

PC12 cells (clone 251; [54]) were maintained, propagated, and transfected as described [37]. In co-transfection experiments, 30–70 μg of each of the corresponding plasmids were added to an electroporation cuvette. Cells were plated onto 25-mm polylysine-coated glass coverslips and used 24–48 h later for experiments. Mouse embryonic chromaffin cells were isolated, maintained, and plated onto coverslips essentially as previously described [55].

### Plasmids.

Plasmids encoding the following constructs were used for overexpression under the control of the CMV promoter: human Neuropeptide-Y, C-terminally fused to enhanced GFP (NPY-GFP [33]); N-terminally myc-tagged Munc18–1 [27]; syntaxin 1A, C-terminally fused to a monomeric variant of enhanced GFP (syntaxin 1A–mGFP, [51]); and a mutant form of a syntaxin 1A (carrying the mutations L165A and E166A) C-terminally fused to monomeric-enhanced GFP (open syntaxin 1A–mGFP). For the latter construct, using PCR, we amplified the coding sequence of the open mutant of syntaxin from pBOB5.1-cmyc–open syntaxin 1A [51] and inserted it into the pEGFP-N1 vector (Clontech, Mountain View, California, United States) carrying a point mutation resulting in a monomeric variant of GFP [51].

### Antibodies.

Mouse monoclonal antibodies were used for detection of syntaxin 1A/B (HPC-1 [56]) and the myc-tag (CRL-1729 ATCC). For Munc18–1, a rabbit anti-serum was raised against recombinant full-length Munc18–1 attached to a T7-tag. As secondary antibodies, goat anti–rabbit-Cy3/Cy5 or goat anti–mouse-Cy3 (Dianova, Hamburg, Germany) were used.

### Recombinant proteins.

Constructs encoding for syntaxin 1A (1–262), SNAP-25 (with cysteines in the linker region replaced by serines), synaptobrevin 2 (1–96), endobrevin (1–71), and VAMP4 (1–117) were as previously described [46]. BoNT/C1 (which cleaves syntaxin between residues 254 and 255), cleavage-inactive BoNT/C1 (E230A), BoNT/E (cleaving SNAP-25 between residues 180 and 181), and TeNT (cleaving synaptobrevin 2 between residues 76 and 77) light chains cloned in the pQE3 vector (Qiagen, Erkrath, Germany) were gifts from Heiner Niemann and Thomas Binz (Medizinische Hochschule Hannover, Hannover, Germany) and subcloned using PCR into the pET28a vector (Novagen/Merck Biosciences, Darmstadt, Germany). The encoding sequences of the resulting constructs were verified by DNA sequencing. All proteins were expressed as His_6_-tagged fusion proteins and purified by Ni^2+^-agarose. In addition, for SNAREs and botulinum neurotoxins, the His-tags were removed by thrombin cleavage, and proteins were purified by ion-exchange chromatography. Cleavage activity of the toxins was tested using as substrates either recombinant synaptobrevin 2 (1–96) and SNAP-25 or syntaxin 1 on plasma membrane sheets [42]. Toxins were dialyzed against KGlu buffer (120 mM KGlu, 20 mM KAc, 20 mM HEPES [pH 7.2]) containing 1 mM DTT.

### Immunofluorescence on membrane sheets.

Cells grown on glass coverslips were sonicated in ice-cold KGlu buffer containing 2 mM EGTA, 4 mM MgCl_2_, and 2 mM ATP, applying a 100-ms ultrasound pulse. They were then either directly fixed in 4% paraformaldehyde in PBS (150 mM NaCl, 10 mM Na_2_HPO_4_, 10 mM NaH_2_PO_4_ [pH 7.2]) for 1 h at room temperature or incubated with KGlu containing 2 mM EGTA, 4 mM MgCl_2_, 2 mM ATP, 3% BSA (BSA-KGlu), and recombinant proteins when indicated.

After fixation, the paraformaldehyde was quenched for 20 min in PBS containing 50 mM NH_4_Cl, and washed two times in PBS for 10 min each. They were then incubated for 1 h with the primary antibodies (diluted 1:200 to 1:400 in BSA-KGlu). In experiments co-staining for Munc18–1 and syntaxin 1, due to partial exclusion of both stainings, we first incubated for 1 h with the primary antibody raised against Munc18–1, washed several times with PBS, and then incubated for 30 min with the primary antibody-recognizing syntaxin. Subsequently, sheets were washed three times in PBS for 10 min each, followed by a 1-h incubation with secondary antibodies (diluted 1:200 in BSA-KGlu). Membrane sheets were washed three times in PBS for 10 min and then imaged at room temperature in PBS containing 4% of a TMA-DPH–saturated PBS solution (Molecular Probes, Eugene, Oregon, United States), in order to judge the continuity and shape of the membrane imaged. In experiments comparatively studying immunostaining intensities, membrane sheets were selected and first imaged in the TMA-DPH channel, and then imaged in the Cy3 channel. In experiments analyzing the co-localization of Munc18–1 domains with secretory granules, during imaging, 0.2-μm TetraSpeck beads (Molecular Probes) were added and allowed to attach to the glass surface, acting as a spatial reference for lateral shifts occurring during filter change.

### Fluorescence microscopy and quantitation of fluorescence intensity.

Membrane sheets were imaged using a Zeiss Axiovert 100 TV fluorescence microscope with a 100× 1.4 NA plan achromate objective (Zeiss, Oberkochen, Germany). For imaging, we used either a back-illuminated frame transfer CCD camera (2× 512 × 512-EEV chip, 13 × 13 μm pixel size; Princeton Scientific Instruments, Monmouth Junction, New Jersey, United States) with a 1.6× Optovar magnifying lens or a back-illuminated CCD camera (512 × 512-NTE chip, 24 × 24 μm pixel size; Princeton Scientific Instruments) with a 2.5× Optovar magnifying lens. TMA-DPH fluorescence was detected using Zeiss filter set 02 (excitation filter G 365, BS 395, and emission LP 420). The following filter sets were used for the detection of GFP, Cy3, and Cy5 fluorescence (all from AHF Analysentechnik AG, Tübingen, Germany): for GFP, excitation filter BP 480/40, BS 505, and emission filter BP 527/30; for Cy3, excitation filter BP 525/30, BS 550 LP, and emission filter BP 575/30; for Cy5, excitation filter BP 620/60, BS 660LP, and emission filter BP 700/75. Images were acquired and analyzed using the program Metamorph (Universal Imaging Corporation, Downingtown, Pennsylvania, United States). Comparative quantitation of fluorescence intensity was done as previously described [42], analyzing for each independent experiment and data point 30–80 membrane sheets when PC12 cells, and 30–50 membrane sheets when mouse chromaffin cells were used. In Figure 1C, the fluorescence trace was fitted to a double-exponential decay according to the formula f(x) = 101.09 AU + 37.44 AU× e^(−x × ln(2)/5 s)^ + 235.88 AU × e^(−x × ln(2)/1,000 s)^. For co-localization analysis, overlays of the red and green channels were generated in Metamorph (Universal Imaging Corporation) using Tetra Speck-fluorescent beads (Molecular Probes) as a spatial reference. Linescans 25-pixel long and 3-pixel broad were placed on the centers of fluorescent granules, and image intensities in the red and green channels were exported and plotted in Sigma Plot 2002 (SPSS Science Software, Chicago, Illinois, United States). Graphs as shown in Figure 2D were printed out and secretory granules were rated to co-localize with a Munc18–1 domain when the peaks of both maxima coincided to within two pixels (corresponds to 192 nm). From ten membrane sheets, 174 granules were analyzed, resulting in 85% co-localizing granules. Background co-localization was determined after mirroring one channel (148 granules from ten membrane sheets analyzed), and background co-localization was subtracted as previously described [42], resulting in a specific co-localization of 70%.

## Abbreviations

BoNT: botulinum neurotoxin
PC12: phaeochromocytoma
SM: Sec1/Munc18
